# The association between parenthood and health: A comparison of people in same-sex and different-sex relationships

**DOI:** 10.1016/j.ssmph.2024.101685

**Published:** 2024-05-28

**Authors:** Yuxuan Jin, Deni Mazrekaj

**Affiliations:** a Netherlands Interdisciplinary Demographic Institute (NIDI) - KNAW/University of Groningen, Lange Houtstraat 19, 2511 CV, Den Haag, the Netherlands; b Department of Sociology, Utrecht University, Padualaan 14, 3584 CH, Utrecht, the Netherlands; c Nuffield College, University of Oxford, New Road, OX1 1NF, Oxford, UK; d Leuven Economics of Education Research, KU Leuven, Naamsestraat 69, 3000, Leuven, Belgium

**Keywords:** Parents, Same-sex relationships, LGBTQI+, Mental health, Physical health, Children, Minority stress, Alcohol, Smoking

## Abstract

Understanding social inequalities in parental health is crucial for family functioning and child development. Theoretically, the double burden of parenting and minority stress may lead to the negative association between parenthood and health outcomes being stronger for people in same-sex relationships. Moreover, drawn from the social control process and the compensation mechanism, the negative association between parenthood and health risk behaviors may become stronger for people in same-sex relationships. Yet, empirical evidence on parental health disparities between parents in same- and different-sex relationships is limited. Using linear and logistic regression models, coarsened exact matching, and entropy balancing on Dutch data between 2008 and 2021 (196 people in same-sex relationships and 6948 people in different-sex relationships), we investigate the relationship between parenthood and three health outcomes (self-rated health, physical health, and mental health) and two health risk behaviors (smoking and heavy episodic drinking). We find that parents on average are less likely to experience heavy episodic drinking than non-parents. The association between parenthood and health does not differ between people in same-sex and different-sex relationships.

## Highlights

• Understanding social inequalities in parental health is crucial for family functioning and child development.
• Empirical evidence that compares people in same-sex and different-sex relationships on the association between parenthood and health is limited.
• Using a nationally representative sample in the Netherlands, the findings indicate that parents on average are less likely to experience heavy episodic drinking than non-parents.
• Results suggest that the association between parenthood and health does not differ between people in same-sex and different-sex relationships.

Parenthood is a transforming life event that may affect parental well-being (Nomaguchi & Milkie, 2020; Umberson et al., 2010). Despite the joy of having a baby, parents tend to have worse mental and physical health than non-parents (Evenson & Simon, 2005; McLanahan & Adams, 1989; Simon, 2008). Same-sex couples may experience worse health consequences of parenthood as they may not enjoy full parental rights and face many challenges on the way to parenthood. Out of 43 European countries, 41 countries provide insemination with donor sperm to different-sex couples, but only 19 countries provide it to female same-sex couples (Fincham, 2021). In addition, same-sex parents may have to navigate a heteronormative context, which assumes that a family comprises two different-sex parents and their children (Charlton, 2022). Same-sex parents also need to put more effort to cope with prejudice and discrimination in their daily lives (Titlestad & Robinson, 2019).

In this article, we investigate whether the association between parenthood and health differs between people in same-sex and different-sex relationships. A few studies comparing the health of parents with non-parents among gay and lesbian couples found no differences in mental health between same-sex parents and non-parents (Assink et al., 2022; Shenkman & Shmotkin, 2014; Zhang et al., 2021). Two studies found that gay fathers smoked more than childless gay men (Zhang et al., 2021), and lesbian mothers drank less alcohol than childless lesbian women (Everett et al., 2022). Although these studies found some evidence for the association between parenthood and health among same-sex couples, they did not include different-sex parents and non-parents as a comparison group, which is important for understanding the underlying theoretical mechanisms. For instance, Simon et al. (2022) found that sexual minority parents were more likely to have worse physical health than childless sexual minority people. However, this health disparity could stem from general parental stress instead of unique minority parental stresses.

To address these research gaps, our study considers all four groups: same-sex parents, same-sex non-parents, different-sex parents, and different-sex non-parents. The empirical evidence on whether the association between parenthood and health differs between people in same-sex and different-sex relationships is limited. To date, two papers come closest to our study. Denney, Gorman, & Barrera (2013) found that the association between the presence of children at home and self-rated general health did not differ between same-sex couples and different-sex married couples. In another paper, Denney, Onge, Gorman, & Krueger (2020) further studied the association between parenthood and health behaviors (smoking and drinking alcohol) by separating people in different-sex married relationships and people in same-sex relationships.

We contribute to their studies in two ways. First, we broaden the evidence to relatively more health-related outcomes than typically seen in prior literature. Specifically, our article is the first study to compare people in same-sex and different-sex relationships on the association between parenthood and mental and physical health. The findings on general self-rated health by Denney, Gorman, & Barrera (2013) may not apply to more specific health outcomes. Although they found that parents have better self-rated health than non-parents, many other studies found that parents are more likely to report worse mental health, such as depression, than non-parents (McLanahan & Adams, 1989; Evenson & Simon, 2005; Nomaguchi & Milkie, 2003). Second, we develop a new theoretical framework to understand how parenthood shapes health risk behaviors (smoking and drinking alcohol) differently for people in same-sex and different-sex relationships. To answer our research question, we use a multi-wave household survey with a nationally representative sample in the Netherlands collected between 2008 and 2021. The data contain 196 people in same-sex relationships and 6948 people in different-sex relationships.

## 1 Background

### 1.1 Parenthood, health, and same-sex parents: previous empirical evidence

Previous studies tend to find that compared to non-parents, parents had worse mental health (McLanahan & Adams, 1989; Evenson & Simon, 2005; Nomaguchi & Milkie, 2020), physical health (Saurel-Cubizolles et al., 2000; Schytt & Hildingsson, 2011; Simon & Caputo, 2019), and overall health. Research on health risk behaviors tends to draw opposite conclusions for smoking and drinking alcohol. Most studies showed that parents had less alcohol consumption (Levy et al., 2018; Patrick et al., 2020) and smoking (Bricard et al., 2017; Tian et al., 2017) than non-parents, even among older parents with adult children (Kendig et al., 2007). Note that most previous studies on parenthood and health were situated in the U.S. context, which may differ from other countries. Specifically, the Netherlands is a country with relatively generous family policies (Evertsson et al., 2020). Those family policies, such as paid parenting leave, may reduce parental stress and thus cancel the negative effect of parenthood on health outcomes. A similar finding has been found in the work on parenthood and happiness (Glass et al., 2016).

Most previous studies on parenthood and health did not consider whether same-sex parent families may differ from different-sex parent families. A few recent cross-sectional studies compared same-sex parents with same-sex non-parents and found no differences in mental health between same-sex parents and non-parents (Assink et al., 2022; Shenkman & Shmotkin, 2014; Zhang et al., 2021). Opposite to the findings from the cross-sectional research, two longitudinal studies in the United States found that same-sex adoptive parents experienced more anxiety and depressive symptoms after they adopted the first child (Goldberg & Smith, 2008, 2011). In terms of physical health, Simon et al. (2022) found that sexual minority parents were more likely to report diseases, such as heart diseases and sleep disorders, than childless sexual minority people. In addition to health outcomes, a few studies examined smoking and drinking alcohol behaviors. Zhang et al. (2021) found that cisgender gay fathers were more likely to smoke than childless gay men, but not lesbian women. Everett et al. (2022) found that lesbian parents had more problem drinking behaviors than non-parents, whereas lesbian parents and non-parents had similar levels of smoking.

Only two studies examined whether the relationship between parenthood and health differs by relationship type (Denney, Gorman, & Barrera, 2013, Denney, Onge, Gorman, & Krueger, 2020). Denney, Gorman, & Barrera, (2013) found that the positive association between the presence of children at home and self-rated health did not differ between same-sex cohabiting couples and different-sex married couples. After separating men and women, their study found that the positive association between the presence of children at home and self-rated general health was stronger for same-sex female couples compared to different-sex married women, but not for same-sex male couples. Denney, Onge, Gorman, & Krueger, (2020) found that parenthood was associated with a lower probability of smoking for different-sex married women, but not for same-sex coupled women. This study also found that parenthood was associated with a lower probability of heavy drinking for both different-sex married women and same-sex coupled women. However, this study did not further investigate whether these negative associations between parenthood and health risk behaviors significantly differ between different-sex married women and same-sex coupled women. We build on their research by providing the first study that compares people in same-sex and different-sex relationships on the association between parenthood and mental and physical health.

### 1.2 The Dutch context

The Netherlands was the first country to legalize same-sex marriage in 2001 and has acknowledged parental rights for same-sex couples for over twenty years. Same-sex couples can become parents through various paths, including joint adoption, artificial insemination, and altruistic surrogacy. Both same-sex parents could take relatively generous parental leaves just as different-sex parents (Kaufman et al., 2022). Apart from laws and family policies, the Netherlands also has tolerant attitudes towards same-sex parenting. For instance, attitudes towards same-sex couples’ rights have become more favorable over time in the Netherlands. The percentage of people who were in favor of gay and lesbian couples having the right to adopt children increased from 77% to 85% between 2016 and 2020 (ESS ERIC, 2023). The Dutch government has also introduced new amendments to existing laws to support the rights of same-sex couples. Namely, altruistic surrogacy was legally available to same-sex male couples from 2019 onwards (Evertsson et al., 2020).

## 2 Theoretical perspectives

### 2.1 Stress process model and unique stressors of same-sex parents for health outcomes

We use the stress process model and minority stress model to investigate the relationship between parenthood and health outcomes of individuals in different-sex and same-sex relationships. The basic premise of the stress process model is that experiencing negative life events and persistent strains are two major sources of stress (Pearlin, 1989). Parents may experience parental role strain when they find it hard to fulfill their duties and responsibilities as parents (Nomaguchi & Milkie, 2020). When children are minors, parents need to put a lot of time and money into doing many childcare tasks, such as feeding, dressing, or taking care of their children. The parental role strain may continue throughout different life stages of parenthood. As children become adolescents, parents may face even more stress as they are involved in more childcare activities, such as attending meetings at schools, picking up and dropping off children from school and home, and disciplining their children to behave well (Meier et al., 2018). Even after their children become adults, parent-child conflict may still become a stressor that increases the psychological distress of parents in their middle to later life (Reczek & Zhang, 2016). These economic, time, emotional, and physical childcare demands could make parents feel stressed and in turn cause mental health problems.

Physical health is also an important stress outcome in the stress theory. McEwen used the term “allostatic load” to explain the association between stress and physical health from the biomedical perspective ( McEwen, 2006). When people face a stressful situation, their heart rate and blood pressure may increase because the body creates chemical mediators (e.g., catecholamines) to help people cope with the situation. Long-term exposure to chronic stress can lead to persistent wear and tear on the body's systems, including elevated heart rate and blood pressure, and the stress may eventually result in physical disorders such as strokes. This phenomenon was coined “allostatic load.” Therefore, parenting stress may not only affect mental health but also physical health, particularly sleep deprivation, fatigue, and headache. Under the stress of childcaring, sleep deprivation is a common health problem, especially for parents with young children (McCann, 2008). Parents also often feel persistent fatigue because of heavy tasks and chores of childcare (Nelson et al., 2014). Headache is another important physical symptom when people are under stress (Schramm et al., 2015). Prior work has shown that parenting stress is likely to increase the number of days people have a headache monthly (Johnson, 2019). Moreover, parents may become more physically vulnerable and have less energy and leisure time to take care of themselves when they have more parenting stress. Therefore, these stressors will further lead to poorer health outcomes, including overall health, mental health, and physical health. Hence, we formulate the following hypothesis:

Hypothesis 1 Parents are more likely to report worse health outcomes, including self-rated health (H1a), mental health (H1b), and physical health (H1c), than non-parents.

Drawn upon the stress process model, the minority stress model points out that sexual minority people have two sources of unique minority stressors, namely external stressors (e.g., prejudice and discrimination) and internal stressors (such as the internalization of negative social attitudes towards sexual minorities). The unique minority stress could further lead to worse health for sexual minority people than straight people (Meyer, 1995). The stress process model and the minority stress model suggest that same-sex parents could have a double burden from general parental stress and unique minority stress. On the one hand, same-sex parents may have more general parental stress than same-sex non-parents. On the other hand, same-sex parents may experience more unique minority stressors than different-sex parents. Same-sex parents need to handle prejudice and stigma toward same-sex families. For instance, opponents of same-sex families often questioned whether same-sex parents could raise children as well as different-sex parents (Stone, 2019).

The heteronormative context could also be an important and unique stressor, which assumes that a family consists of a father, a mother, and their children. Many qualitative studies showed that same-sex parents often felt excluded when other people used language that did not apply to same-sex parents, such as asking “who is dad and who is mum?” (Charlton, 2022; Titlestad & Robinson, 2019). Same-sex parents may also internalize this heteronormative assumption as a stressor; they may start to question themselves about whether they are capable of being good parents (Charlton, 2022). Peleg and Hartman (2019) found that it was not enough for lesbian mothers to show that they are good parents like others, but they had to prove themselves to be perfect parents. Moreover, these parents were seen as role models of the LGBTQ+ community, adding further weight to their responsibilities. Consequently, the expectations and perceptions surrounding same-sex parents can become a considerable burden to bear. These minority parental stressors may create a more stressful environment when same-sex couples become parents, which amplifies the negative effect of parenthood on health outcomes. Hence, we formulate the following hypothesis:

Hypothesis 2 The negative association between parenthood and health outcomes, including self-rated health (H2a), mental health (H2b), and physical health (H2c), may be stronger for people in same-sex relationships than for those in different-sex relationships.

### 2.2 Social control theory and unique compensation process of same-sex parents for health risk behaviors

The stress process model suggests that people may turn to coping behaviors to manage stress. Coping behaviors may include engaging in health risk behaviors, such as smoking and drinking alcohol. However, the stress process theory may be less appropriate for explaining why parents might engage in fewer risky health behaviors than non-parents as found in previous empirical studies (Bricard et al., 2017; Levy et al., 2018; Patrick et al., 2020; Tian et al., 2017). Hence, we provide another new theoretical perspective to conceptualize why the association between parenthood and health risk behaviors differs between people in same-sex and different-sex relationships by combining the social control theory and compensation theory.

Social control is a mechanism referring to social relationships that have both direct and indirect influence on health behavior (Umberson, 1987). Direct social control suggests that an external person can monitor and regulate another's health behavior directly. In a parent-child relationship, parents may often instruct children to have a healthy lifestyle and punish them if children drink or smoke before adulthood (Ennett et al., 2001; Lindsay et al., 2006). On the other hand, children could also regulate their parents' health behavior. For instance, Backett-Milburn and Jackson (2012) found that young children are already concerned about their parental health problems, such as alcohol misuse. Direct social control from adult children may be even stronger. Adult children may be more concerned about the health problems of their parents as their parents grow older. As shown in earlier literature (Reczek et al., 2014), adult daughters often attempted and encouraged their parents to improve their exercise and diet.

The indirect social control process refers to people being self-motivated to monitor and regulate their health behavior because of the internalized norms and meanings of a social role (Reczek et al., 2014; Umberson, 1987). When people become parents, they may internalize the responsibility and obligation of a parent. Parents may feel responsible to provide a healthier environment for their children. Therefore, parents may in turn start to promote their healthy behavior on purpose. For instance, parents may smoke less when they notice that children may suffer from secondhand smoke if they smoke at home. In addition to the motivation to create a healthy environment, parents may also reduce smoking and drinking alcohol as they would like to be healthier to take care of their children (Reczek et al., 2014). Thus, we formulate the following hypothesis:

Hypothesis 3 Parents are less likely to smoke (H3a) and experience heavy episodic drinking (H3b) than non-parents.

The mechanism of social control may differ between people in same-sex and different-sex relationships according to compensation theory. The compensation theory is widely used in psychology. Compensation refers to a process to overcome losses or deficits through investing more time and effort (Dixon & Bäckman, 1995). For same-sex parents who face substantive barriers to parenthood, compensation could mean that these parents channel stressors as motivation to prove that they can raise healthy children as well as different-sex parents (Peleg & Hartman, 2019). This compensation mechanism may further affect parental health behaviors through the indirect social control process. Same-sex parents may have stronger feelings of parental obligation than different-sex parents to reduce smoking and to drink less alcohol given that these behaviors are viewed as bad parenting behaviors by others. For instance, same-sex parents may choose to stay at home with children rather than go out to a bar and drink alcohol to show that they are good parents. Although smoking or drinking alcohol may also happen privately at home, same-sex parents may still reduce these behaviors because a potentially unhealthy child may signal an inability to raise children to others. For instance, if parents smoke at home, children may suffer from passive smoking and have more health problems, such as wheezing and asthma (Burke et al., 2012). With a potentially larger parental obligation, same-sex parents may be more motivated to protect their children from unhealthy environments. Therefore, when same-sex couples become parents, this unique compensatory process and social control theory may lead to the following hypothesis:

Hypothesis 4 The negative association between parenthood and smoking (H4a) and heavy episodic drinking (H4b) may be stronger for people in same-sex relationships than those in different-sex relationships.

## 3 Data and methods

### 3.1 Sample construction

We use the Longitudinal Internet Studies for the Social Sciences (LISS) panel administered by CentERdata (Tilburg University, the Netherlands) from 2008 to 2021. The LISS panel includes a probability sample of households drawn from the population registers in the Netherlands, which comprises more than 6500 households and more than 15,000 individuals. A longitudinal survey is fielded in the panel every year, covering a large variety of domains including family, health, work, education, income, housing, time use, political views, and personality (Scherpenzeel & Das, 2010). We use three modules from the LISS panel data, namely family and household, health, and background variables. The primary advantage of LISS data is that they allow for simultaneous comparisons based on the type of relationship respondents are in (same-sex vs. different-sex) as well as parental status (yes vs. no). Such data that include four groups (“same-sex parents, same-sex non-parents, different-sex parents, and different-sex non-parents”) are rare. Moreover, compared to most previous literature on same-sex parenthood and health that used non-probability samples (e.g., Zhang et al., 2021), another advantage of LISS data is that they are a nationally representative sample drawn from population registers.

We combine all 14 waves of the LISS data from 2008 to 2021, resulting in a sample of 15,720 individuals. We further restrict the sample in five ways. First, we exclude those respondents who did not participate in family and households or health modules, which resulted in a sample of 13,884 individuals. Second, as our study compares people in different-sex and same-sex relationships, we restrict the sample to individuals who are in a relationship at the time of the interview. This restriction results in 10,748 individuals who are in a relationship. Third, we only choose one wave for each respondent rather than use the information of each respondent every year as in longitudinal data. The reason why we transform the longitudinal design to the cross-sectional design is that less than ten respondents in same-sex relationships transitioned to parenthood from 2008 to 2021, hindering us from fully exploiting the longitudinal design of the data. Nonetheless, these data are useful as they allow us to examine a range of specific health indicators for the first time, such as mental health and physical health. To capture the most recent information of the respondents, we select the latest wave that those respondents participated in.

Fourth, we exclude five parents with no children alive to exclude the effect of children's deaths on parental health. Fifth, as the information on migration background is only available after 2010, we exclude respondents who were surveyed before 2011. Then, we use listwise deletion to remove observations with missing information on parenthood status, health indicators, and other control variables. The proportion of missing values for each variable is below 1% except the household income. The proportion of missing values for the household income is 9.7%. In robustness checks, we do not use migration background as a control variable, which could keep respondents who were interviewed before 2011. We also use multiple imputation to impute the missing values of the household income (Allison, 2000). Results are very similar to our main findings (see Online Supplement Tables S4 and S5). The final sample includes 7144 individuals in a relationship, among which 196 individuals are in same-sex relationships and 6948 individuals are in different-sex relationships.

### 3.2 Variables

#### 3.2.1 Same-sex relationship

We create a variable given a value of 1 if the respondent is in a same-sex relationship and 0 if the respondent is in a different-sex relationship. The survey asks the respondents to report their own gender and their partner's gender. The response options for the two questions were “male” and “female.” If the respondents have the same gender as their partners, they are considered to be in a same-sex relationship.^1^ If the respondents have a different gender from their partners, they are considered in a different-sex relationship. Specifically, our paper only examines people in same-sex and different-sex relationships at the individual level rather than same-sex and different-sex couples at the couple level.

#### 3.2.2 Dependent variables

Dependent variables include self-rated health, mental health, physical health problems, smoking status, and heavy episodic drinking.

Self-rated health is measured by the question: “How would you describe your health, generally speaking?“. The five answer options included: (1) poor, (2) moderate, (3) good, (4) very good, and (5) excellent. Following previous literature (Lagos, 2019), we recode self-rated health as a dichotomous variable to ensure sufficient variation. We classify “poor” and “moderate” as bad health, and “good”, “very good”, and “excellent” as good health. As a robustness check, we code self-rated health as a 5-point continuous variable, obtaining similar results (available upon request).

Mental health is measured by five questions: “How did you feel during this past month: (a) I felt very anxious, (b) I felt so down that nothing could cheer me up, (c) I felt calm and peaceful, (d) I felt depressed and gloomy, and (e) I felt happy.” Each question has options on a 6-point scale including never, seldom, sometimes, often, mostly, and continuously. These five questions belong to the Mental Health Inventory (MHI-5) questionnaire that is often used to measure depressive symptoms (Rumpf et al., 2001). The scale of these five questions in the LISS panel has a high reliability with a 0.857 Cronbach's alpha. We calculate the scores of the scale following the established guidelines (Ware et al., 1993). The score for each question is from 1 to 6. As the first, second, and fourth questions are related to negative feelings, we reverse their scores. For each respondent, we sum up the scores of five questions ranging from 5 to 30. We transform raw scale scores into a continuous variable ranging from 0 to 100. A higher score indicates a better level of mental health.

Physical health problems are measured by three questions that ask respondents whether they regularly suffer from a type of health problem. These health problems include headache, fatigue, and sleeping problems, which are closely related to parenting stress (Johnson, 2019, McCann, 2008, Nelson et al., 2014). Each question has yes (1) and no (0) options. We count the three items and create a continuous variable ranging from 0 to 3 to indicate the number of physical health problems that respondents suffer from regularly. A higher score indicates more physical health problems. We also recode physical health problems as a dichotomous variable by measuring whether the respondent has any physical health problems or not as a robustness check. The results are similar to our main findings (see Online Supplement S7).

Smoking status is measured by a dichotomous variable that indicates whether the respondent smoked at that moment or not. We create this binary variable based on two questions. The first question asks the respondents whether they had ever smoked. The respondent who said no is considered someone who did not smoke at that moment. For those respondents who had ever smoked, the following question is asked: “Do you smoke now?“. We code those respondents who answered “no, I stopped” as respondents who did not smoke at that moment. Conversely, those respondents who answered yes are considered people who smoked at that moment.

Heavy episodic drinking is measured by the number of drinks on the heaviest drinking day during the past seven days. An advantage of the questionnaire is that it asks the number of drinks for each type of drink (e.g., beer, strong beer, strong spirits or liquors, sherry or martini, wine, alcohol pops) and each type of serving (e.g., a regular glass, a half liter glass, cans or bottles, and small cans or bottles). We convert all types of drinks into standard drinks based on the quantity of drinks and average alcohol content in the Netherlands.^2^ In the Netherlands, a standard drink contains 10g of alcohol. Following prior work (Crutzen & Giabbanelli, 2014), we categorize respondents as people who have experienced heavy episodic drinking if the number of drinks on the heaviest drinking day is at least four for women, and at least five for men.

As a robustness check, we use the frequency of alcohol consumption as another measurement of drinking behavior. The frequency of alcohol consumption is measured by the question: “How often did you have a drink containing alcohol over the last 12 months?“. The question includes 8 options: (1) almost every day, (2) five or six days per week, (3) three or four days per week, (4) once or twice a week, (5) once or twice a month, (6) once every two months, (7) once or twice a year, (8) not at all over the last 12 months. Few respondents are in some categories. For instance, only nine respondents in same-sex relationships drink alcohol five or six days per week. To ensure sufficient variation, we collapse eight items into a 4-point scale following previous studies (Poelen et al., 2005): (1) seldom or never (items 7 and 8), (2) a few times a month (items 5 and 6), (3) a few times a week (items 3 and 4), and (4) often or always (items 1 and 2). Results are similar to our findings on heavy episodic drinking (see Online Supplement Tables S1 and S2).

#### 3.2.3 Independent variable

The independent variable is parenthood status. We measure parenthood status as people who have at least one child who is alive at the time of the interview. The question text in the LISS panel questionnaire on whether the respondent has any children changed throughout the 14 waves. From Wave 1 to Wave 7, the item was “Have you had any children? With this we mean biological children (gotten with your partner or someone else) as well as stepchildren, adoptive children, and foster children.” and it asks respondents to include deceased children. As we have already removed respondents who only have deceased children, we can code respondents who said yes as parents and respondents who said no as non-parents. After Wave 8, the item changed to “Did you ever have any children? With this we mean biological children (gotten with your partner or someone else) as well as stepchildren, adoptive children, and foster children.” and it tells respondents to exclude deceased children. We code respondents who say yes as parents and respondents who say no as non-parents. Note that a limitation of how parenthood is measured is that the LISS panel did not include any information on whether parents actually raised the child after it was born. Thus, it is possible that respondents only birthed children but did not raise them.

#### 3.2.4 Control variables

Following previous studies (Mazrekaj et al., 2020; Zhang et al., 2021), we control for age, sex (0 is man, 1 is woman), the educational level of respondents (lower than secondary education, secondary education, and university education), urbanicity (0 is not urban, 1 is urban), marital status (0 is not married, 1 is married), cohabitation (0 is not living together, 1 is living together), monthly net household income in 1000 euros, migration background (0 is Dutch background, 1 is non-Dutch background). We also add indicators for the survey year as dummy variables to control for the potential impacts of time and year-specific sociopolitical events.

#### 3.2.5 Analytic strategy

We first present descriptive statistics by parenthood status (parents and non-parents) and relationship type (same-sex and different-sex relationships). To compare parents with non-parents within each relationship type, we use a two-tailed *t*-test for continuous variables and a Chi-square test for categorical variables. Next, we run regression models for people in same-sex and different-sex relationships. We use linear regression models for continuous outcomes including mental health and physical health problems. We use logistic regression models for binary outcomes including self-rated health, smoking status, and heavy episodic drinking. We present average marginal effects (AMEs) for logistic regression models (Mood, 2010). To test whether the association between parenthood and health differs between people in same-sex and different-sex relationships, we add the interaction term between parenthood status and relationship type in our models. Following the strategy by Mize (2019), we test the differences in AMEs of parenthood between people in same-sex and different-sex relationships to determine the size and significance of interaction terms. As the cases could be dependent (both members of a household are sometimes in the dataset as unique observations), we cluster standard errors at the household level.

To assess whether the relatively small size of people in same-sex relationships (n = 196) has sufficient statistical power to detect a significant effect, we perform a posthoc power analysis using G*power version 3.1.9.7 with α = 0.05 (Faul et al., 2007). This analysis shows that our sample of people in same-sex relationships has sufficiently high power (99.2%) to detect moderate effect sizes (f^2^ = 0.10), and relatively high power (87.6%) to detect small effect sizes (f^2^ = 0.05) using nine predictors. Unfortunately, we do not have sufficient power to differentiate between male and female same-sex relationships. We further plot the sensitivity power analysis curve suggested by Lakens (2022) to detect the power of the sample of people in same-sex relationships. The sensitivity power analysis curve is used to examine how large an effect size a study could detect with a desired power. As shown in Fig. S1 in the Online Supplement, our sample of people in same-sex relationships can detect relatively small effects. For instance, Fig. S1 shows that the sample is sensitive to the effects of Cohen's d = 0.04 with 80% power.

As a robustness check (see Online Supplement Table S3 from Panel A to Panel E), we use Poisson regression models to estimate the association between parenthood status and the number of physical health problems. We also estimate models using coarsened exact matching (CEM) to reduce the bias in the treatment effect because of the imbalance in size between the comparison and the control group (Blackwell et al., 2009; Iacus et al., 2012). Iacus et al. (2012) found that CEM performs better than regression-based methods and propensity score matching (PSM). Finally, we also use entropy balancing (Hainmueller, 2012). Unlike CEM which discards observations that cannot be matched, entropy balancing has the advantage that it does not discard any treated units. Our results are robust to these additional models. All analyses are conducted in Stata 15.1.

## 4 Results

### 4.1 Descriptive findings

We first present the descriptive findings by comparing individuals in different-sex and same-sex relationships (see Online Supplement Table S6). In line with previous studies (Jaspers & Verbakel, 2012), people in same-sex relationships are less likely to be parents in the Netherlands: 38 percent of people in same-sex relationships and 76 percent of people in different-sex relationships are parents. Around 80 percent of respondents in both same-sex and different-sex relationships report good self-rated health. People in same-sex relationships are significantly more likely to report worse mental and physical health than people in different-sex relationships. People in different-sex and same-sex relationships have similar levels of heavy episodic drinking. As for smoking status, people in same-sex relationships are 7 percentage points more likely to smoke than people in different-sex relationships.

To understand the socioeconomic and demographic characteristics of the sample more thoroughly, Table 1 presents descriptive statistics by parenthood status (parents and non-parents) and relationship type (same-sex and different-sex relationships). First, we compare parents with non-parents within each relationship type. For both relationship types, parents are more likely to be older, lower educated, married, and living together. It appears that parents are more likely to be male, live in rural areas, have a Dutch background, and have higher net household income for people in different-sex relationships than people in same-sex relationships.

**Table 1:** Descriptive statistics.

	People in different-sex relationships		People in same-sex relationships	
	*Parents*	*Non-parents*	*Parents*	*Non-parents*
Self-rated health (1 is good)	0.80	0.87^a^	0.73	0.84
Mental health (0–100)	75.57	71.66^a^	72.91	68.93
Physical health problems	0.69	0.71	0.91^c^	0.94^d^
Smoking status (1 is yes)	0.12	0.16^a^	0.13	0.24^d^
Heavy episodic drinking (1 is yes)	0.15	0.28^a^	0.15	0.27^b^
Age	56.63	36.93^a^	55.15	44.37^b^^d^
Sex (1 is woman)	0.52	0.57^a^	0.53	0.41^d^
Educational level				
lower than secondary education	0.28	0.18^a^	0.31	0.15^b^
secondary education	0.61	0.60^a^	0.59	0.69^b^
university education	0.11	0.22^a^	0.11	0.17^b^
Marital status (1 is married)	0.80	0.29^a^	0.71	0.26^b^
Cohabitation (1 is living together)	0.92	0.68^a^	0.92	0.64^b^
Urban	0.79	0.84^a^	0.81	0.88
Net household income (in 1000 EUR)	3527.83	3353.43^a^	3738.11	3372.89
Migration Background (1 is non-Dutch)	0.15	0.19^a^	0.19	0.19
Number of respondents	5270	1678	75	121

a Among people in different-sex relationships, the coefficient of parents is significantly different from the baseline coefficient of non-parents at the 5 percent level.

b Among people in same-sex relationships, the coefficient of parents is significantly different from the baseline coefficient of non-parents at the 5 percent level.

c Among parents, the coefficient of people in same-sex relationships is significantly different from the baseline coefficient of people in different-sex relationships at the 5 percent level.

d Among non-parents, the coefficient of people in same-sex relationships is significantly different from the baseline coefficient of people in different-sex relationships at the 5 percent level.

Second, we compare people in different-sex and same-sex relationships within parents and non-parents. Among parents, significance tests do not show any significant differences between people in different-sex and same-sex relationships for all socioeconomic and demographic characteristics. Among non-parents, significance tests also do not show significant differences for most variables. We find that people in same-sex relationships are more likely to be older and male. Descriptive comparisons as in Table 1, however, do not control for possible differences in observed characteristics between the four groups.

### 4.2 Association between parenthood and health outcomes and behaviors

**Fig. 1:**
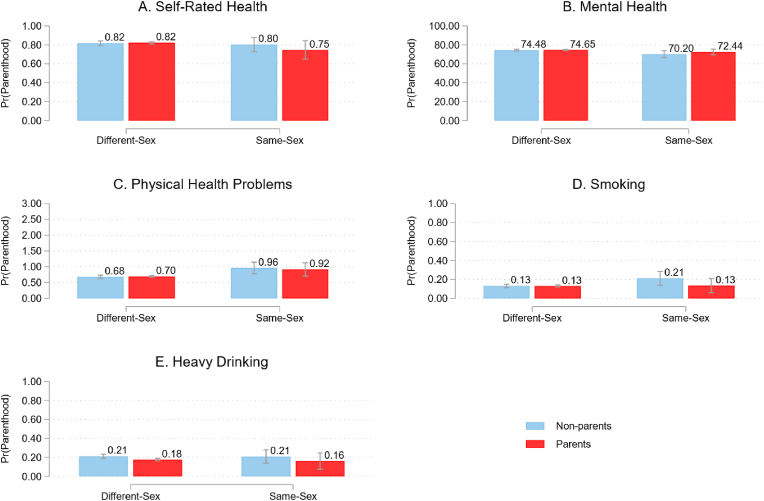
Predicted probabilities/values by parenthood and same-sex relationship type (vertical bars indicate 95% CIs). Results on self-rated health, smoking, and heavy episodic drinking are from binary logistic regression models; the predicted probabilities are presented. Results on mental health and physical health are from linear regression models; the predicted values are presented.

Table 2 presents the results from regression models that predict three health outcomes and two health risk behaviors. Each variable has two columns that present the results without control variables and with control variables, and with the interaction term between parenthood status and same-sex relationship. We present all results on the probability scale to facilitate the interpretation (Mize, 2019; Mood, 2010). Following the suggestions on examining interaction effects by Mize (2019), we first present the average predicted probabilities for binary variables and average predicted values for continuous variables by parenthood and relationship type (Fig. 1). Then we present the average marginal effects (AMEs) of parenthood by relationship type and contrasts on whether these effects differ between people in different-sex and same-sex relationships (Table 3).

**Table 2:** Results from regression models predicting health outcomes and health behaviors.

	Self-rated health	Self-rated health	Mental health	Mental health	Physical health	Physical health	Smoking	Smoking	Heavy episodic drinking	Heavy episodic drinking
	(1a)	(1b)	(2a)	(2b)	(3a)	(3b)	(4a)	(4b)	(5a)	(5b)
Parenthood status	−0.07***	0.001	4.05***	0.25	−0.03	0.01	−0.05***	−0.004	−0.13***	−0.04**
	(0.01)	(0.01)	(0.47)	(0.57)	(0.03)	(0.03)	(0.01)	(0.01)	(0.01)	(0.01)
Same-sex relationship		−0.04		−3.46**		0.26***		0.05		−0.01
		(0.03)		(1.27)		(0.07)		(0.03)		(0.03)
Age		−0.00***		0.12***		−0.00		−0.00***		−0.00***
		(0.00)		(0.01)		(0.00)		(0.00)		(0.00)
Sex (1 is woman)		−0.01		−3.65***		0.36***		−0.04***		−0.11***
		(0.01)		(0.35)		(0.02)		(0.01)		(0.01)
Education (ref. < sec. edu.)										
secondary education		0.06***		2.05***		−0.08**		−0.05***		0.03*
		(0.01)		(0.51)		(0.03)		(0.01)		(0.01)
university education		0.09***		1.76*		−0.15***		−0.10***		0.04**
		(0.02)		(0.71)		(0.04)		(0.01)		(0.02)
Marital status (1 is married)		−0.02		1.43*		0.03		−0.04**		−0.06***
		(0.01)		(0.56)		(0.03)		(0.01)		(0.01)
Cohabitation (1 is living together)		0.02		0.87		−0.11*		−0.01		−0.01
		(0.02)		(0.70)		(0.04)		(0.01)		(0.01)
Net household income (in 1000 EUR)		0.02***		0.92***		−0.05***		−0.01***		0.01***
		(0.00)		(0.13)		(0.01)		(0.00)		(0.00)
Urban (ref. rural)		−0.03**		−0.89		0.07*		0.00		0.00
		(0.01)		(0.50)		(0.03)		(0.01)		(0.01)
Migration background		−0.05***		−3.50***		0.11***		0.02*		−0.06***
(1 is non-Dutch)		(0.01)		(0.60)		(0.03)		(0.01)		(0.01)
Survey year	No	Yes	No	Yes	No	Yes	No	Yes	No	Yes
Observations	7144	7144	7144	7144	7144	7144	7144	7144	7144	7144

*Notes:* (1) Results on self-rated health, smoking, and heavy episodic drinking are from binary logistic regression models; average marginal effects are presented. (2) Results on mental health and physical health are from linear regression models; the coefficient estimates are presented. (3) Standard errors are between parentheses. (4)* p < 0.05, ** p < 0.01, *** p < 0.001 (two-tailed tests).

**Table 3:** Average marginal effects (AMEs) of parenthood and tests for the moderating role of a same-sex relationship (N = 7144).

Outcomes	Relationship type	AME of parenthood	Contrast
A. Self-rated health	(1a) Different-Sex	0.004	−0.06 (0.06)
		(0.01)	
	(1b) Same-Sex	−0.05	
		(0.06)	
B. Mental health	(2a) Different-Sex	0.17	2.07 (2.45)
		(0.58)	
	(2b) Same-Sex	2.23	
		(2.41)	
C. Physical health problems	(3a) Different-Sex	0.02	−0.06 (0.15)
		(0.03)	
	(3b) Same-Sex	−0.05	
		(0.14)	
D. Smoking	(4a) Different-Sex	−0.001	−0.08 (0.06)
		(0.01)	
	(4b) Same-Sex	−0.08	
		(0.06)	
E. Heavy episodic drinking	(5a) Different-Sex	−0.04**	−0.02 (0.06)
		(0.01)	
	(5b) Same-Sex	−0.05	
		(0.06)	

*Notes:* (1) Results on self-rated health, smoking, and heavy drinking are from binary logit models; results on mental health and physical health are from linear regression models. All models include control variables: age, sex, educational level, marital status, cohabitation, urban, monthly net household income in euros, migration background, and survey year. (2) Standard errors are between parentheses. (3) * p < 0.05, ** p < 0.01, *** p < 0.001 (two-tailed tests).

Parents report significantly lower levels of self-rated health than non-parents as shown in Column 1a of Table 2. After we control for other variables (see column 1b of Table 2), the AME of parenthood status becomes close to zero for self-rated health. Thus, parents do not have worse self-rated health than non-parents, which does not support our Hypothesis 1a. In Panel A of Table 3, we test whether AMEs of parenthood differ between people in different-sex and same-sex relationships. The contrast is not significant suggesting that the association between parenthood and self-rated health does not differ between people in same-sex and different-sex relationships.

In addition to self-rated health, column 2a and column 3a in Table 2 show that parents tend to have significantly better mental health than non-parents without controlling for any variables, but not for physical health problems. The coefficients of parenthood status for mental health and physical health are both very close to zero and not significant after controlling for other variables (see column 2b and column 3b of Table 2). In particular, we find that the estimated coefficient for mental health drops from 4.05 to 0.25, which indicates that control variables may account for most of the association between parenthood status and mental health. All the coefficients of the control variables have the expected sign. Our results show that older age, higher education, income, and marriage are significantly associated with better mental health. Women and people from a non-Dutch background are more likely to have worse mental health. In terms of the interaction terms (see Panel B and Panel C of Table 3), the contrasts of AMEs are not significant suggesting that the associations between parenthood status and mental health and physical health do not differ between people in same-sex and different-sex relationships. Overall, results show that parenthood status is not significantly associated with any health outcomes, therefore not supporting our Hypothesis 1. Similarly, our Hypothesis 2 is also not supported given that the association between parenthood and all health outcomes does not differ between people in different-sex and same-sex relationships.

In terms of health risk behaviours, we find that parents are significantly less likely to smoke than non-parents (see column 4a of Table 2). After we add control variables in the models, however, the coefficient is very close to zero and not significant anymore (see column 4b of Table 2). The AMEs of parenthood do not differ between people in different-sex and same-sex relationships (see Panel D of Table 3). As for the heavy episodic drinking, we find that parents are 4 percentage points less likely to have experienced heavy episodic drinking than non-parents after adding control variables (see column 5b of Table 2). The contrast of AMEs in Panel E of Table 3 is not significant suggesting that the association between parenthood status and heavy episodic drinking does not significantly differ between people in different-sex and people in same-sex relationships. In conclusion, the results support our Hypothesis 3b, but not Hypothesis 3a. Parents are significantly less likely to experience heavy episodic drinking than non-parents, but they are just as likely to smoke. Finally, the results do not support Hypothesis 4a nor Hypothesis 4b. The association between parenthood and health risk behaviors (both smoking and heavy episodic drinking) is not significantly different between people in same-sex and different-sex relationships.

## 5 Discussion

Using data from the Netherlands, our study examined whether the association between parenthood and health differs between people in same-sex and different-sex relationships. Contrasting with most previous studies in other countries, our study shows that parenthood status is not associated with any health outcomes (self-rated health, mental health, and physical health) in the Netherlands. We only find that parents are less likely to experience heavy episodic drinking than non-parents, although they do not adjust their smoking behavior. In terms of the comparison between people in same-sex and different-sex relationships, we do not find that the association between parenthood on the one hand and health outcomes and health risk behaviors on the other significantly differs between these two groups.

Our results indicate that parents do not have worse mental health, physical health, and overall self-rated health than non-parents. The findings on health outcomes are not in line with some prior research (e.g., Nomaguchi & Milkie, 2003; Saurel-Cubizolles et al., 2000). Two reasons may explain these findings. First, the rewards of raising children may reduce the negative effects of childbearing stress (Nomaguchi, 2012; Umberson et al., 2010). For instance, older adult children may become an important source of social support to parents rather than a stressor (Simon & Caputo, 2019). Second, most prior research is situated in the United States, a country with high costs of raising children (Glass et al., 2016). The Netherlands has relatively generous family policies to reduce childbearing stress for parents (Evertsson et al., 2020). Using the Netherlands Kinship Panel Study, Keizer et al. (2010) also did not find that transition to parenthood influenced parental well-being (life satisfaction, loneliness, and positive affect), but it reduced the negative affect of parents.

Nonetheless, it is surprising to find that parenthood status is not associated with smoking but is significantly associated with the reduction of the probability of heavy episodic drinking. Given the social control process perspective, parents should both drink and smoke less than non-parents as they regulate their health behaviors on purpose to provide a healthier environment for their children. For these contrasted findings, the opportunity perspective derived from social role theory may provide another explanation. According to social role theory, parenthood is related to a more structural life as parents need to take responsibility to take care of their children. Thereby, parents have fewer opportunities to allocate time and resources for heavy episodic drinking than non-parents. For instance, Paradis (2011) found that the reduction in drinking at bars could partly explain the negative association between parenthood and drinking alcohol. But this opportunity perspective does not necessarily apply to smoking, which depends less on time and location.

The findings on the comparison of people in same-sex and different-sex relationships in terms of health outcomes are consistent with previous studies. Denney, Gorman, & Barrera, (2013) did not find that the positive association between the presence of children and self-rated health significantly differs between same-sex cohabiting couples and different-sex married couples. These findings have two implications for the minority stress theory. First, the stress for Dutch same-sex parents may be less than our expectations from the minority stress theory. The Netherlands has a relatively tolerant environment towards same-sex parents. According to the European Social Survey in 2018, 85.6% of respondents in the Netherlands supported that gay male and lesbian couples should have the same rights to adopt children as straight couples (ESS ERIC, 2023). Second, minority stress theory also needs to take the resilience of same-sex parents into account (Prendergast & MacPhee, 2018). Despite experiencing stigma and discrimination when raising children, same-sex parents may have unique protective factors that buffer against these negative effects of minority stressors. For instance, previous studies showed that same-sex parents may receive social support from the LGBTQ+ community during the transition to parenthood (Leal et al., 2021) and their children generally do well on various outcomes, such as school, mental health, labor, and behavioral outcomes (Mazrekaj et al., 2020, 2022; Mazrekaj & Jin, 2023; Palmaccio et al., 2023). The additional support may help same-sex parents cope with those unique minority stressors.

Our result on smoking and heavy episodic drinking does not support our hypothesis that given the compensatory psychological process, the negative association between parenthood and smoking and heavy episodic drinking should be stronger for people in same-sex relationships. One may argue that same-sex parents may not necessarily smoke and drink less alcohol to prove themselves as good parents as these health behaviors are private and other people may not see them. But our results suggest that parents were more likely to reduce drinking alcohol rather than smoking compared to non-parents. This finding indicates that parents may have fewer opportunities to allocate time for heavy episodic drinking because drinking alcohol is more dependent on time and location. Drinking alcohol is also more likely to be public and involves other people compared to smoking. Another reason why the compensation mechanism does not work could be that same-sex parents may have fewer incentives to increase their parental efforts when they live in a relatively tolerant environment with fewer minority stressors as is the case in the Netherlands. Mazrekaj et al. (2020) found that the positive association between same-sex parents and the school outcomes of their children was stronger before the legalization of same-sex marriage in 2001 than afterward in the Netherlands. This finding suggests that the compensation mechanism becomes weaker over time. As our data are collected after the legalization of same-sex marriage, same-sex parents may feel no need to put more effort than different-sex parents to reduce their smoking and heavy episodic drinking. Finally, although the expectation to be a good parent and raise a healthy child may motivate same-sex parents to reduce health risky behaviors, proving to be a good parent is also a unique minority-related parenting stressor (Peleg & Hartman, 2019). To cope with this minority stressor, same-sex parents may smoke more and drink more alcohol. Thus, this minority-related parenting stressor may cancel out the hypothesized positive effect of the compensation mechanism.

Overall, our study contributes to previous literature by showing that the association between parenthood and health does not differ between people in same-sex and different-sex relationships. Most previous studies on parenthood and health did not consider same-sex parent families (see Nomaguchi & Milkie, 2020 for a review of the literature). Although several recent studies focus on the impact of parenthood on health and well-being among same-sex couples (Assink et al., 2022; Shenkman & Shmotkin, 2014; Zhang et al., 2021), the empirical evidence that considers all four groups simultaneously (same-sex parents, same-sex non-parents, different-sex parents, and different-sex non-parents) is very limited. This limitation hinders us from understanding to what extent same-sex families fare well compared with different-sex families.

Our findings have important implications for society and policy. First, practitioners (such as clinicians) may help same-sex couples reduce worries about potential parenting stress and minority stress from discrimination, and stereotypes when they hope to become a parent (Shenkman & Shmotkin, 2014). Our results suggest that parenthood is likely not a harmful event for parental health no matter if you are in a same-sex or different-sex relationship in the Netherlands. Second, our findings add to prior evidence that supports the ability of same-sex parents to take care of their children (Farr & Vazquez, 2020). Same-sex couples appear to have a similar ability to cope with parenting challenges as different-sex couples. Hence, policymakers should further remove barriers that prevent same-sex parents from becoming parents.

This study is not without limitations. Despite using a range of models, the reader should be cautious in interpreting our results as causal. For instance, healthier people may be more likely to become parents. This selection may undermine the negative effects of parenthood on health outcomes. Moreover, the selection bias into parenthood may differ between same-sex and different-sex couples. Same-sex couples may have more family planning on their way to parenthood as they often need more preparation to become a parent, for instance when using fertility treatments (Mazrekaj et al., 2020). This selection bias may undermine the gap regarding the negative effect of parenthood on health between same-sex and different-sex couples. To reduce the bias of selection into same-sex parenthood, future studies could use longitudinal data and fixed effects models to study the impact of the transition into same-sex parenthood on health and well-being. The longitudinal administrative register data linked with health information could be a suitable option, which covers the population of a whole country over decades. Moreover, our study could not separate the sample by sex because of the small sample size of people in same-sex relationships, nor could we observe the respondents’ sexual orientation. For instance, bisexual people may display significantly different behavior than gay male and lesbian individuals (Jaspers, Mazrekaj, & Machado, 2024). Lastly, our study could not further investigate the mechanisms between parenthood and health as the LISS data do not include information on stress and social support. Future research could examine how social support buffers the negative effects of parental stress on health for same-sex couples.

## 6 Conclusion

Our study provided new evidence on whether the association between parenthood and health differs between people in same-sex and different-sex relationships, particularly mental health and physical health for the first time. In conclusion, we found that the association between parenthood and health does not differ between people in same-sex and different-sex relationships. Our study adds to the previous literature on family structure, health and well-being, and same-sex parenting. Our findings highlight that same-sex and different-sex parents are likely to fare well in a context that provides similar family policies support and parental rights. We encourage future studies to use longitudinal data to further study the causal effect of the transition to same-sex parenthood on health and well-being.

## Financial disclosure statement

Deni Mazrekaj acknowledges funding from the European Union's Horizon Europe program under grant agreement 101129146 (EFFEct).

## Ethical statement

This research has been approved by the Ethics Review Board of the Faculty of Social and Behavioural Sciences at Utrecht University (Reference number 22–1820). The authors declare that they have no known competing financial interests or personal relationships that could have appeared to influence the work reported in this paper.

## CRediT authorship contribution statement

**Yuxuan Jin:** Writing – review & editing, Writing – original draft, Visualization, Validation, Software, Methodology, Investigation, Formal analysis, Data curation, Conceptualization. **Deni Mazrekaj:** Writing – review & editing, Writing – original draft, Visualization, Validation, Supervision, Software, Methodology, Investigation, Formal analysis, Data curation, Conceptualization.

## Declaration of competing interest

The authors declare that they have no known competing financial interests or personal relationships that could have appeared to influence the work reported in this paper.

## Data Availability

The authors do not have permission to share data.

## Footnotes

1 We use terms “same-sex” and “different-sex” throughout the article for consistency. Although the LISS panel questions asked about own and partner's gender, the response options were only “male” and “female” before February 2022, which are categorically sex-designation variables. We do not know whether respondents answered these two questions referring to sex assigned at birth, current gender identity, or registered sex on the official ID documentation. Moreover, LISS panel data do not include a direct measure of sexual orientation. We only identify people in same-sex and different-sex relationships based on the above questions.

2 The LISS panel survey did not provide the official guideline. We follow the guideline from the European Social Survey Wave 11 in the Netherlands European Social Survey. (2022). ESS has a similar module on similar type of drinks and provides the formula to calculate the grams of alcohol.
