# Supplementary material for: The association between parenthood and health: A comparison of people in same-sex and different-sex relationships
Source: SSM Popul Health. 2024 May 28;26:101685. doi: 10.1016/j.ssmph.2024.101685 (PMC11179624; doi:10.1016/j.ssmph.2024.101685)

**ONLINE SUPPLEMENT**

to article in

SSM-Population Health

**The Association Between Parenthood and Health: A Comparison of People in Same-Sex and Different-Sex Relationships**

^1^Yuxuan Jin (correspondence author, email: yuxuan.jin@rug.nl): Netherlands Interdisciplinary Demographic Institute (NIDI) - KNAW/University of Groningen, Lange Houtstraat 19, 2511 CV, Den Haag, The Netherlands; Department of Sociology, Utrecht University, Padualaan 14, 3584 CH, Utrecht, The Netherlands.

^2^Deni Mazrekaj (email: d.mazrekaj@uu.nl): Department of Sociology, Utrecht University, Padualaan 14, 3584 CH, Utrecht, The Netherlands; Nuffield College, University of Oxford, New Road, OX1 1NF, Oxford, UK; Leuven Economics of Education Research, KU Leuven, Naamsestraat 69, 3000, Leuven, Belgium.

| Table S1. Average Marginal Effects (AMEs) From Ordinal Logistic Models Predicting Frequency of Alcohol Consumption | | | | |
| --- | --- | --- | --- | --- |
|  | seldom or never | a few times a month | a few times a week | often or always |
| Parenthood | 0.06^***^ | 0.02^***^ | -0.03^***^ | -0.05^***^ |
|  | (0.01) | (0.00) | (0.00) | (0.01) |
| Controls^a^ | Yes | Yes | Yes | Yes |
| Observations | 7144 | 7144 | 7144 | 7144 |

^a^Controls include age, sex, educational level, marital status, cohabitation, urban, monthly net household income in euros, migration background, and survey year.

** p < 0.05, ** p < 0.01, *** p < 0.001 (two-tailed tests)*

| Table S2: Average Marginal Effects (AMEs) of Parenthood and Tests For Moderating Role of Same-Sex Relationship For Frequency of Alcohol Consumption (N = 7144) | | | | |
| --- | --- | --- | --- | --- |
|  | Relationship type |  | AME of parenthood | Contrasts |
| seldom or never | (1a) Different-Sex |  | 0.06^***^ | -0.06  (0.04) |
|  |  |  | (0.01) |  |
|  | (1b) Same-Sex |  | -0.00 |  |
|  |  |  | (0.04) |  |
| a few times a month | (2a) Different-Sex |  | 0.02^***^ | -0.02  (0.02) |
|  |  |  | (0.00) |  |
|  | (2b) Same-Sex |  | -0.00 |  |
|  |  |  | (0.02) |  |
| a few times a week | (3a) Different-Sex |  | -0.03^***^ | -0.03  (0.02) |
|  |  |  | (0.00) |  |
|  | (3b) Same-Sex |  | 0.00 |  |
|  |  |  | (0.02) |  |
| often or always | (4a) Different-Sex |  | -0.06^***^ | -0.06  (0.05) |
|  |  |  | (0.01) |  |
|  | (4b) Same-Sex |  | 0.00 |  |
|  |  |  | (0.05) |  |

** p < 0.05, ** p < 0.01, *** p < 0.001 (two-tailed tests)*

| Table S3. Results from Different Methods Predicting Health Outcomes and Behaviors. | | | | | | | | | | | | | | | | | | | | |
| --- | --- | --- | --- | --- | --- | --- | --- | --- | --- | --- | --- | --- | --- | --- | --- | --- | --- | --- | --- | --- |
| *Panel A. Self-rated health* | | | | | | | | | | | | | | | | | | | | |
|  | (1) | | (2) | | | | | (3) | | | | (4) | | |  | | | |  | |
| Parenthood | 0.00 | | 0.01 | | | | | -0.01 | | | | 0.02 | | |  | | | |  | |
|  | (0.02) | | (0.02) | | | | | (0.04) | | | | (0.03) | | |  | | | |  | |
| Parenthood × |  | | -0.06 | | | | |  | | | | -0.07 | | |  | | | |  | |
| Same-sex rel. |  | | (0.07) | | | | |  | | | | (0.06) | | |  | | | |  | |
| Controls^a^ | Yes | | Yes | | | | | Yes | | | | Yes | | |  | | | |  | |
| Methods^b^ | CEM | | CEM | | | | | Entropy | | | | Entropy | | |  | | | |  | |
| Observations | 4,012 | | 4,012 | | | | | 7,144 | | | | 7,144 | | |  | | | |  | |
| *R*^2^ | 0.075 | | 0.075 | | | | | 0.084 | | | | 0.085 | | |  | | | |  | |
| Pseudo *R*^2^ |  | |  | | | | |  | | | |  | | |  | | | |  | |
| *Panel B. Mental health* | | | | | | | | | | | | | | | | | | | |  |
|  | | | (1) | | | | | | (2) | | | | | (3) | | | | (4) | | |
| Parenthood | | | 0.54 | | | | | | 0.50 | | | | | 0.89 | | | | -0.03 | | |
|  | | | (1.17) | | | | | | (1.22) | | | | | (1.31) | | | | (1.00) | | |
| Parenthood × Same- | | |  | | | | | | 0.58 | | | | |  | | | | 1.82 | | |
| sex rel. | | |  | | | | | | (2.66) | | | | |  | | | | (2.29) | | |
| Controls^a^ | | | Yes | | | | | | Yes | | | | | Yes | | | | Yes | | |
| Methods^b^ | | | CEM | | | | | | CEM | | | | | Entropy | | | | Entropy | | |
| Observations | | | 4,012 | | | | | | 4,012 | | | | | 7,144 | | | | 7,144 | | |
| *R*^2^ | | | 0.087 | | | | | | 0.087 | | | | | 0.101 | | | | 0.101 | | |
| *Panel C. Physical health problems* | | | | | | | | | | | | | | | | | | | | |
|  | | (1) | | | | (2) | | | | | (3) | | (4) | | | (5) | | | (6) | |
| Parenthood | | 0.02 | | | | 0.02 | | | | | -0.01 | | -0.01 | | | -0.07 | | | -0.07 | |
|  | | (0.04) | | | | (0.05) | | | | | (0.07) | | (0.07) | | | (0.08) | | | (0.06) | |
| Parenthood × Same- | |  | | | | -0.09 | | | | |  | | -0.03 | | |  | | | 0.01 | |
| sex rel. | |  | | | | (0.16) | | | | |  | | (0.17) | | |  | | | (0.14) | |
| Controls^a^ | | Yes | | | | Yes | | | | | Yes | | Yes | | | Yes | | | Yes | |
| Methods^b^ | | Poisson | | | | Poisson | | | | | CEM | | CEM | | | Entropy | | | Entropy | |
| Observations | | 7,144 | | | | 7,144 | | | | | 4,012 | | 4,012 | | | 7,144 | | | 7,144 | |
| *R*^2^ | |  | | | |  | | | | | 0.090 | | 0.090 | | | 0.093 | | | 0.093 | |
| *Panel D. Smoking status* | | | | | | | | | | | | | | | | | | | | |
|  | | | | (1) | | | (2) | | | | (3) | | (4) | | |  | | |  | |
| Parenthood | | | | 0.01 | | | 0.02 | | | | -0.08^**^ | | -0.05^*^ | | |  | | |  | |
|  | | | | (0.03) | | | (0.03) | | | | (0.03) | | (0.02) | | |  | | |  | |
| Parenthood × Same-sex | | | |  | | | -0.11 | | | |  | | -0.07 | | |  | | |  | |
| rel. | | | |  | | | (0.06) | | | |  | | (0.05) | | |  | | |  | |
| Controls^a^ | | | | Yes | | | Yes | | | | Yes | | Yes | | |  | | |  | |
| Methods^b^ | | | | CEM | | | CEM | | | | Entropy | | Entropy | | |  | | |  | |
| Observations | | | | 4,012 | | | 4,012 | | | | 7,144 | | 7,144 | | |  | | |  | |
| *R*^2^ | | | | 0.052 | | | 0.053 | | | | 0.096 | | 0.098 | | |  | | |  | |
| Pseudo *R*^2^ | | | |  | | |  | | | |  | |  | | |  | | |  | |
| *Panel E. Heavy episodic drinking* | | | | | | | | | | | | | | | | | | | | |
|  | |  | | |  | | | | |  | | |  | | | |  | |  | |
| Parenthood | | (1) | | | (2) | | | | | (3) | | | (4) | | | |  | |  | |
|  | | -0.05 | | | -0.05 | | | | | -0.06 | | | -0.04 | | | |  | |  | |
| Parenthood × Same- | | (0.03) | | | (0.03) | | | | | (0.03) | | | (0.02) | | | |  | |  | |
| sex rel. | |  | | | -0.01 | | | | |  | | | -0.04 | | | |  | |  | |
| Controls^a^ | |  | | | (0.06) | | | | |  | | | (0.06) | | | |  | |  | |
| Methods^b^ | |  | | |  | | | | |  | | |  | | | |  | |  | |
| Observations | | CEM | | | CEM | | | | | Entropy | | | Entropy | | | |  | |  | |
| *R*^2^ | | 4,012 | | | 4,012 | | | | | 7,144 | | | 7,144 | | | |  | |  | |
| Pseudo *R*^2^ | | 0.088 | | | 0.088 | | | | | 0.086 | | | 0.086 | | | |  | |  | |
| *Note:* Standard errors are between parentheses.  ^a^Controls include age, sex, educational level, marital status, cohabitation, urban, monthly net household income in euros, migration background, and survey year.  ^b^CEM stands for coarsened exact matching. Entropy stands for entropy balancing.  ** p < 0.05, ** p < 0.01, *** p < 0.001 (two-tailed tests)* | | | | | | | | | | | | | | | | | | | | |

| Table S4. Results from linear regression models predicting health outcomes and behaviors that do not control migration background. | | | | | | | | | | |  |  |
| --- | --- | --- | --- | --- | --- | --- | --- | --- | --- | --- | --- | --- |
|  | (1a) | (1b) | (2a) | (2b) | (3a) | (3b) | (4a) | (4b) | (5a) | (5b) |  |  |
|  | Self-rated health | Self-rated health | Mental health | Mental health | Physical health | Physical health | Smoking | Smoking | Heavy episodic  drinking | Heavy episodic  drinking |  |  |
| Parenthood status | 0.01 | 0.01 | -0.12 | -0.12 | 0.02 | 0.02 | -0.01 | -0.00 | -0.05^***^ | 0.01 |  |  |
|  | (0.01) | (0.01) | (0.52) | (0.53) | (0.03) | (0.03) | (0.01) | (0.01) | (0.01) | (0.01) |  |  |
| Parents × Same-sex rel. |  | -0.06 |  | 0.16 |  | 0.00 |  | -0.05 |  | 0.01 |  |  |
|  |  | (0.06) |  | (2.22) |  | (0.13) |  | (0.05) |  | (0.05) |  |  |
| Controls^a^ | Yes | Yes | Yes | Yes | Yes | Yes | Yes | Yes | Yes | Yes |  |  |
| Observations | 8,491 | 8,491 | 8,491 | 8,491 | 8,491 | 8,491 | 8,491 | 8,491 | 8,491 | 8,491 |  |  |
| *Adjusted R*^2^ | 0.063 | 0.063 | 0.058 | 0.058 | 0.059 | 0.059 | 0.045 | 0.045 | 0.067 | 0.067 |  |  |
| *Note:* Standard errors are between parentheses.  ^a^Controls include age, sex, educational level, marital status, cohabitation, urban, monthly net household income in euros, and survey year.  ** p < 0.05, ** p < 0.01, *** p < 0.001 (two-tailed tests)* | | | | | | | | | | |  |  |

| Table S5. Results from linear regression models predicting health outcomes and behaviors that use multiple imputations of net household income. | | | | | | | | | | |  |  |
| --- | --- | --- | --- | --- | --- | --- | --- | --- | --- | --- | --- | --- |
|  | (1a) | (1b) | (2a) | (2b) | (3a) | (3b) | (4a) | (4b) | (5a) | (5b) |  |  |
|  | Self-rated health | Self-rated health | Mental health | Mental health | Physical health | Physical health | Smoking | Smoking | Heavy episodic  drinking | Heavy episodic  drinking |  |  |
| Parenthood status | 0.01 | 0.01 | 0.16 | 0.07 | 0.01 | 0.02 | 0.00 | 0.00 | -0.05^***^ | -0.05^***^ |  |  |
|  | (0.01) | (0.01) | (0.54) | (0.55) | (0.03) | (0.03) | (0.01) | (0.01) | (0.01) | (0.01) |  |  |
| Parents × Same-sex rel. |  | -0.07 |  | 2.19 |  | -0.06 |  | -0.07 |  | -0.03 |  |  |
|  |  | (0.06) |  | (2.30) |  | (0.13) |  | (0.06) |  | (0.06) |  |  |
| Controls^a^ | Yes | Yes | Yes | Yes | Yes | Yes | Yes | Yes | Yes | Yes |  |  |
| Observations | 7,893 | 7,893 | 7,893 | 7,893 | 7,893 | 7,893 | 7,893 | 7,893 | 7,893 | 7,893 |  |  |
| *R*^2^ | 0.061 | 0.061 | 0.064 | 0.065 | 0.056 | 0.056 | 0.041 | 0.042 | 0.064 | 0.064 |  |  |
| *Note:* Standard errors are between parentheses.  ^a^Controls include age, sex, educational level, marital status, cohabitation, urban, monthly net household income in euros, migration background, and survey year.  ** p < 0.05, ** p < 0.01, *** p < 0.001 (two-tailed tests)* | | | | | | | | | | |  |  |

| Table S6. Descriptive Statistics by Same-sex Relationship. | | | | |
| --- | --- | --- | --- | --- |
|  | People in different-sex relationships | | People in same-sex relationships | |
|  | *Mean* | *S.D.* | *Mean* | *S.D.* |
| Self-rated health (1 is good) | 0.82 | 0.38 | 0.80 | 0.40 |
| Mental health (0-100) | 74.63 | 16.32 | 70.45^a^ | 17.84 |
| Physical health problems | 0.69 | 0.93 | 0.93^a^ | 1.42 |
| Smoking status (1 is yes) | 0.13 | 0.34 | 0.20^a^ | 0.41 |
| Heavy episodic drinking | 0.19 | 0.39 | 0.22 | 0.30 |
| Parenthood status (1 is yes) | 0.76 | 0.43 | 0.38^a^ | 0.49 |
| Age | 51.85 | 17.65 | 48.49^a^ | 16.46 |
| Sex (1 is woman) | 0.53 | 0.50 | 0.46^a^ | 0.50 |
| Educational level |  |  |  |  |
| lower than secondary | 0.25 | 0.44 | 0.21 | 0.40 |
| education |  |  |  |  |
| secondary education | 0.61 | 0.49 | 0.65 | 0.48 |
| university education | 0.13 | 0.34 | 0.14 | 0.36 |
| Marital status (1 is married) | 0.68 | 0.47 | 0.43^a^ | 0.50 |
| Cohabitation (1 is living | 0.86 | 0.34 | 0.75^a^ | 0.43 |
| together) |  |  |  |  |
| Urban | 0.80 | 0.40 | 0.86^a^ | 0.35 |
| Net household income | 3485.71 | 1753.46 | 3512.64 | 1813.92 |
| Migration background (1 is non-Dutch) | 0.16 | 0.36 | 0.19 | 0.39 |
| Number of respondents | 6,948 |  | 196 |  |
| ^a^ The coefficient of people in same-sex relationships is significantly different from the baseline coefficient of people in different-sex relationships in Column 1 at the 5 percent level using a two-tailed t-test for continuous variables and a Chi-square test for categorical variables. | | | | |

| Table S7. Results from linear regression models predicting sleeping problems, fatigue, and headaches. | | | | | | |
| --- | --- | --- | --- | --- | --- | --- |
|  | (1a) | (1b) | (2a) | (2b) | (3a) | (3b) |
|  | Sleeping problems | Sleeping problems | Fatigue | Fatigue | Headache | Headache |
| Parenthood status | -0.02 | -0.02 | 0.02 | 0.02 | 0.01 | 0.01 |
|  | (0.01) | (0.01) | (0.02) | (0.02) | (0.01) | (0.01) |
| Parents × Same-sex rel. |  | -0.02 |  | -0.05 |  | 0.01 |
|  |  | (0.07) |  | (0.07) |  | (0.06) |
| Controls^a^ | Yes | Yes | Yes | Yes | Yes | Yes |
| Observations | 7,107 | 7,107 | 7,107 | 7,107 | 7,107 | 7,107 |
| *Adjusted R*^2^ | 0.044 | 0.044 | 0.032 | 0.032 | 0.049 | 0.049 |
| *Note:* Standard errors are between parentheses.  ^a^Controls include age, gender, educational level, marital status, cohabitation, urban, net household income in Euros monthly, migration background, and survey year.  ** p < 0.05, ** p < 0.01, *** p < 0.001 (two-tailed tests)* | | | | | | |

Figure S1. Sensitivity power analysis


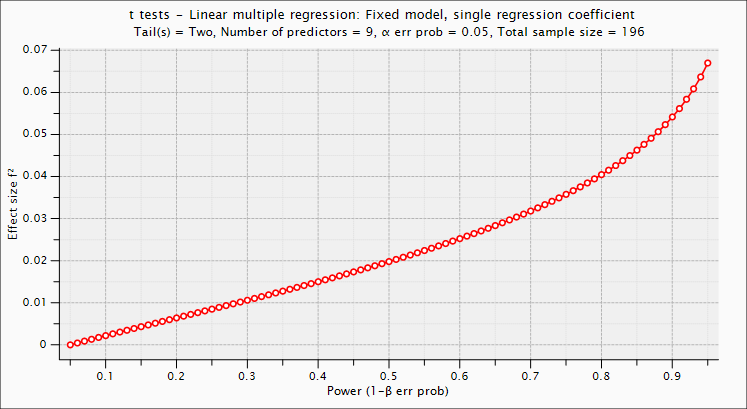

Supplement: Multimedia component 1 [file mmc1.docx]
